# Isotropic incorporation of SPD-5 underlies centrosome assembly in *C. elegans*

**DOI:** 10.1016/j.cub.2015.05.060

**Published:** 2015-08-03

**Authors:** Triin Laos, Gabriela Cabral, Alexander Dammermann

**Affiliations:** Max F. Perutz Laboratories, University of Vienna, Vienna Biocenter (VBC), Dr. Bohr-Gasse 9, A-1030 Vienna, Austria

## Abstract

Centrosomes are important regulators of microtubule organization in animal cells. Within the centrosome, microtubule nucleation and anchorage are mediated by proteins in the pericentriolar material (PCM) that accumulates around centrioles. The spatial organization of the PCM and the contribution of centrioles to its recruitment remain poorly understood. Previous work in the *Drosophila* embryo showed that the key PCM component Cnn specifically incorporates near centrioles, suggesting that centrioles play an ongoing role in PCM assembly [1]. It is currently unclear whether this model holds true in other organisms. Here, we examine PCM dynamics in the *Caenorhabditis elegans* embryo. We find that recruitment of the scaffold component SPD-5 occurs throughout the PCM. Incorporation of additional PCM subunits is therefore not limited to specific nucleation sites near centrioles, which has profound implications for the organization of the PCM lattice and the role of centrioles in centrosome assembly.

## Main Text

Unlike centrioles, the PCM displays no apparent higher-order organization when examined by electron microscopy, although proteins occupy distinct domains based on high-resolution light microscopy [2]. This lack of organization, combined with a lack of hierarchical recruitment dependencies, has made it difficult to delineate assembly pathways as have been developed for centrioles. Instead, a central concept in centrosome assembly is that of a matrix scaffold that forms around centrioles and acts as a docking platform for other proteins that nucleate and organize microtubules [3]. One of the best candidates for a scaffold component is *C. elegans* SPD-5 [4]. Depletion of this protein results in no PCM forming around centrioles and consequently failure of spindle assembly [5]. The dynamics of this protein at centrosomes have not previously been investigated.

To monitor SPD-5 behavior, we generated a GFP fusion under endogenous regulatory sequences by transposon-mediated insertion at a defined chromosomal locus (MosSCI). This transgene was rendered RNAi-resistant by resequencing, enabling specific depletion of the endogenous protein (Figure S1A in the Supplemental Information). Importantly, expression of GFP::SPD-5 fully restored spindle assembly and embryonic viability, confirming functionality of the GFP fusion (Figure S1B,C). Scaffold components are expected to display no exchange with the cytoplasmic pool once incorporated into centrosomes. To examine if this is the case for SPD-5, we performed fluorescence recovery after photobleaching experiments. In order to distinguish cytoplasmic exchange from ongoing recruitment, we first sought to identify a plateau in PCM recruitment. As shown in Figure S2A, SPD-5 levels increase continuously during the first embryonic cell cycle, before declining at mitotic exit. To obtain stable levels of PCM we arrested embryos in metaphase using the proteasome inhibitor clasto-lactacystin-β-lactone (Figure S2A,B). Under these conditions, the PCM regulator AIR-1 displayed rapid exchange with the cytoplasmic pool (Figure S2C). By contrast, no recovery was observed with SPD-5, consistent with the behavior of a scaffold component. The γ-tubulin homolog TBG-1 likewise displayed little cytoplasmic exchange. However, SPD-2, an important regulator of PCM assembly and centriole duplication, displayed substantial exchange, complicating further analysis of the dynamics of this protein.

**Figure 1:**
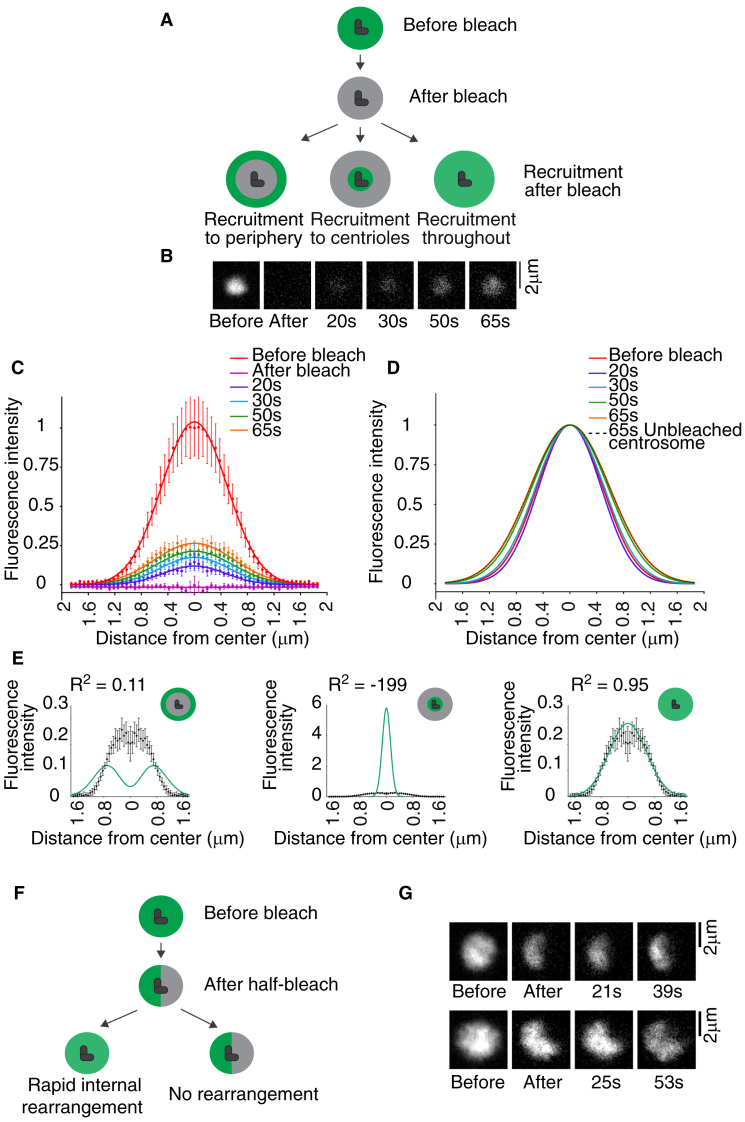
SPD-5 incorporates throughout the volume of the PCM. (A) Schematic of assay to determine spatial dynamics of PCM assembly. Centrosomal GFP signal is bleached during centrosome maturation. Three possible outcomes for the pattern of new protein incorporation are shown. (B) Representative images of GFP::SPD-5 before and after bleaching/maturation. Post-bleach images scaled equivalently. (C,D) GFP::SPD-5 distribution before and after bleaching/maturation. Each profile is an average of >9 centrosomes, normalized to the peak intensity of the pre-bleach signal (C) or the peak intensity at each time point (D). Error bars are the 90% confidence interval for the mean. The slight spread of signal at later time points reflects expansion of the PCM during centrosome maturation. (E) Fit of 65 s timepoint data to predicted fluorescence distribution for each model. (F,G) Bleach marks. created by partial photobleaching of GFP::SPD-5 in metaphase-arrested embryos are maintained over extended time frames. Schematics of potential outcomes (F) and representative images (G), scaled equivalently.

Elegant experiments performed in *Drosophila* embryos have shown that Cnn specifically incorporates near centrioles [1]. To examine whether SPD-5 behaves in a similar manner, we conducted photobleaching experiments on embryos expressing GFP::SPD-5 in mitotic prophase and examined the pattern of recruitment following centrosome maturation. These experiments are facilitated by the large size of centrosomes in the *C. elegans* embryo, around 60 times that in *Drosophila* embryos or vertebrate cultured cells [6]. In principle, additional SPD-5 could be incorporated specifically at centrioles, at the PCM periphery, or throughout the volume of the PCM (Figure 1A). As seen in Figure 1B–E, additional protein is recruited throughout the PCM, closely matching the distribution predicted by model 3. The lack of cytoplasmic exchange of SPD-5 here is essential to unambiguously identify this signal as new protein incorporation. Importantly, there is also no evidence for internal rearrangements or flux of centrosomal SPD-5 which could complicate analysis (Figure 1F,G).

Thus, there is no privileged role for centrioles in scaffold assembly in *C. elegans*. Rather, the PCM expands isotropically by incorporation of additional SPD-5 throughout its volume. Similar results are now reported for Cnn in *Drosophila* somatic cells (see accompanying manuscript by Conduit and Raff). Unlike crystals in solution or typical polymers, the PCM lattice must be able to stretch to accommodate additional subunits. This sponge-like behavior hints at an internal flexibility that would seem at odds with the ability of centrosomes to resist external pulling forces. Cytoplasmic SPD-5 is known to exist in monomeric form [7], which may facilitate incorporation throughout the PCM lattice. While our results do not support a direct role for centrioles in PCM recruitment, centrioles clearly initiate PCM assembly [8]. Kinetic arguments also support an ongoing role controlling the rate of incorporation [9], potentially through centriole-localized regulators such as Plk1 and SPD-2 [4,10]. It will be interesting to see how centrioles exert control over centrosome assembly at a distance and how the *in vitro* properties of SPD-5 and other scaffolding proteins give rise to this unique and dynamic structure.

## Supplemental Information

Document S1. Two Supplemental figures and experimental procedures
