# Supplementary material for: Isotropic incorporation of SPD-5 underlies centrosome assembly in C. elegans
Source: Curr Biol. 2015 Aug 3;25(15):R648–9. doi: 10.1016/j.cub.2015.05.060 (PMC4533240; doi:10.1016/j.cub.2015.05.060)
Supplement: Document S1. Two Supplemental figures and experimental procedures [file mmc1.pdf]

Isotropic Incorporation Of SPD-5 Underlies Centrosome Assembly In *C. elegans*

Triin Laos, Gabriela Cabral, Alexander Dammermann

Inventory of Supplemental Information

Figure S1: GFP:SPD-5 fusion can functionally substitute for the endogenous protein.
Figure S2: SPD-5 is a stably incorporated PCM component.
Supplemental Experimental Procedures
Supplemental References

A

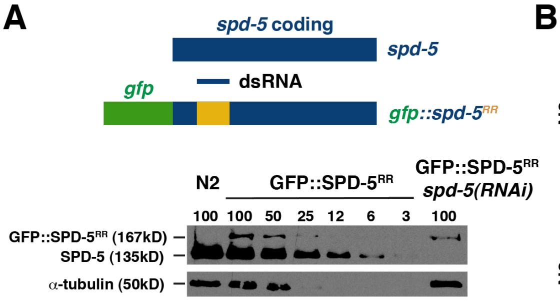

B

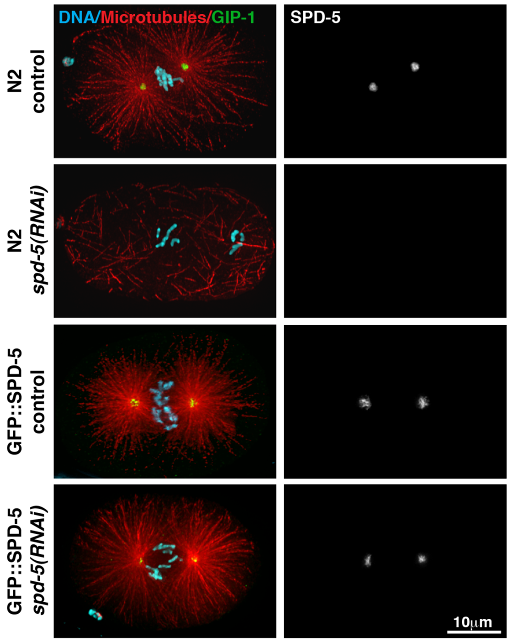

C

Strain, condition	Embryonic viability (%)	n
N2 control	98.8	1289
N2 *spd-5(RNAi)*	0	1305
GFP::SPD-5^{RR} control	99.0	1502
GFP::SPD-5^{RR} *spd-5(RNAi)*	99.1	1505

Figure S1: GFP::SPD-5 fusion can functionally substitute for the endogenous protein. (**A**) Schematic of endogenous SPD-5 and GFP::SPD-5^{RR} transgene. The GFP transgene includes a 600bp segment (yellow) that has been re-encoded for RNAi resistance (RR) by changing the nucleotide sequence without altering the encoded amino acid sequence or codon bias, allowing specific depletion of endogenous SPD-5 using dsRNAs targeting the original sequence. SPD-5 immunoblot comparing extract prepared from GFP::SPD-5^{RR} worms treated with *spd-5* dsRNA to serial dilutions of untreated worms (numbers indicate percent of amount loaded in 100% lane) and wild-type (N2) worms. SPD-5 is depleted to less than 3% of wild-type levels. GFP fusion is unaffected. α-tubulin is included as a loading control. (**B**) Immunofluorescence micrographs of control and *spd-5(RNAi)*-treated wild-type (N2) and GFP::SPD-5^{RR} embryos, stained for DNA, microtubules, and the γ-tubulin complex protein GIP-1 (left), and SPD-5 (right). Depletion of SPD-5 results in failure of PCM recruitment and consequently spindle assembly. Expression of GFP::SPD-5^{RR} restores PCM assembly and spindle formation. (**C**) Embryonic viability of wild-type (N2) and GFP::SPD-5^{RR} worms following control depletion or treatment with *spd-5* dsRNA. Expression of GFP::SPD-5^{RR} fully rescues the lethality associated with SPD-5 depletion.

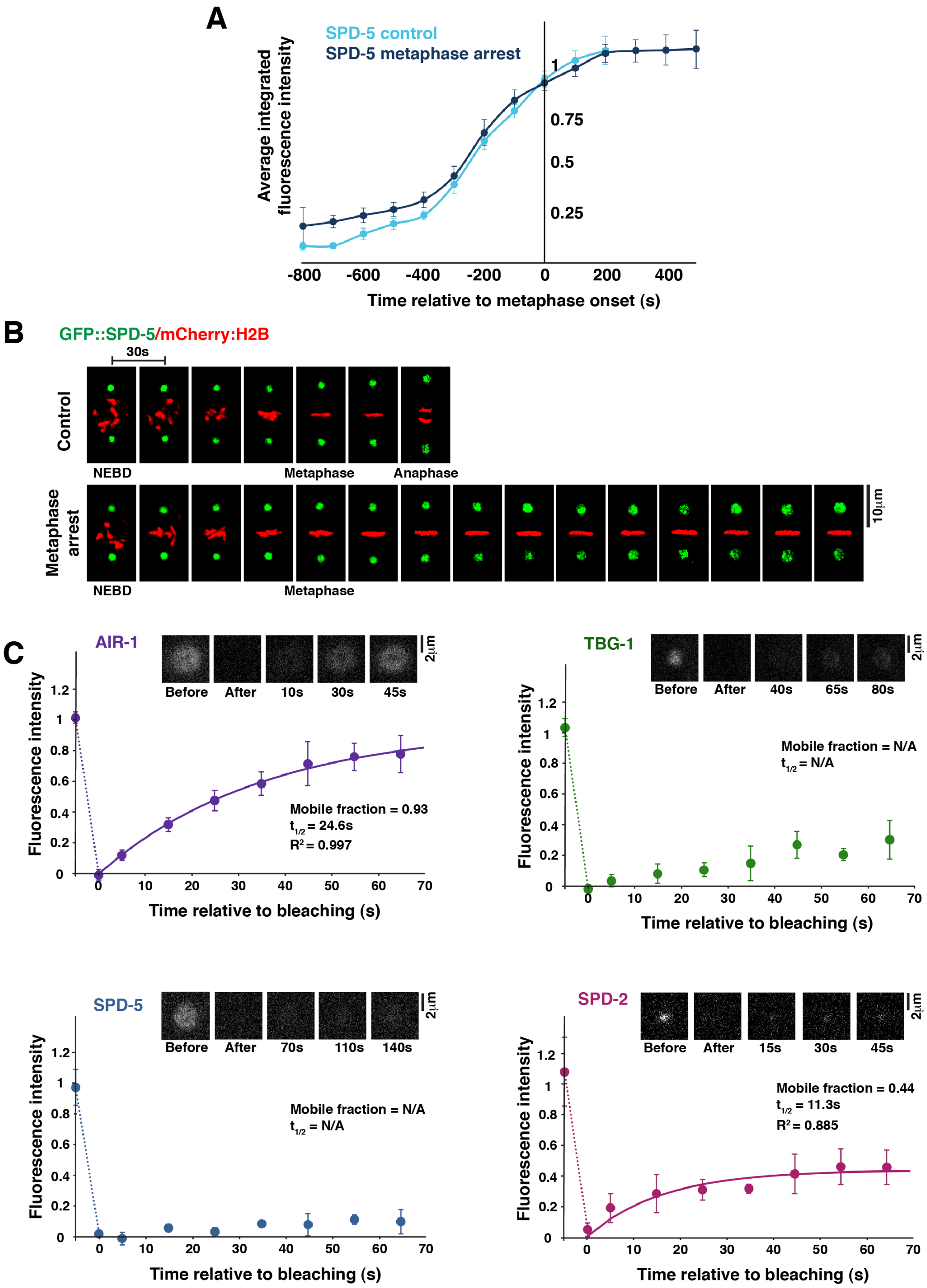

Figure S2: SPD-5 is a stably incorporated PCM component. (**A**) Recruitment profile for GFP::SPD-5 in control (light blue) and 20μM clasto-lactacystin-ß-lactone-treated (dark blue) embryos during the first embryonic cell cycle. Data points are the mean of the normalized GFP intensity measurements collected during the 200s interval centered on that point. PCM levels continuously increase until mitotic exit (~200s after metaphase onset), at which time PCM rapidly disassembles. In metaphase-arrested embryos PCM levels reach a plateau which is maintained for the

duration of the arrest. N=16 embryos (control), 15 (clasto-lactacystin-ß-lactone-treated). (**B**) Still images from timelapse sequences of control and 20μM clasto-lactacystin-ß-lactone-treated embryos expressing GFP::SPD-5 and mCherry::H2B. Proteasome inhibition results in prolonged metaphase arrest (>7-10 minutes). (**C**) Fluorescence recovery after photobleaching (FRAP) profiles and representative images for centrosomal GFP::AIR-1 (Aurora A), TBG-1 (γ-tubulin), SPD-5 and SPD-2 in 20μM clasto-lactacystin-ß-lactone-treated embryos. Photobleaching was performed ~3min 30s into metaphase arrest, at a time when PCM levels have reached a stable plateau (see (**A**)). Images are scaled equivalently. Data points on the graphs are the mean fluorescence intensities at each time point, normalized to the pre-bleach intensity. AIR-1 and SPD-2 displayed rapid exchange with the cytoplasmic pool, for which values for mobile fraction and half-life could be obtained. TBG-1 and SPD-5 displayed no significant exchange. Similar results were obtained in untreated, metaphase-stage embryos (not shown). N=15 embryos (AIR-1), 16 (TBG-1), 17 (SPD-5), 11 (SPD-2). Error bars in (**A**) and (**C**) are the 90% confidence interval for the mean.

SUPPLEMENTAL EXPERIMENTAL PROCEDURES

C. elegans strains and culture conditions

Strains expressing GFP::AIR-1, GFP::SAS-6, mCherry::SPD-2, GFP::SPD-5, GFP::TBG-1, GFP/mCherry::H2B and mCherry::PH$^{PLC\delta 1}$ have been described previously [S1-5]. In generating the GFP::SPD-5 transgene, a 600bp region within exon 3 of *spd-5* was re-encoded for RNAi resistance [S6], enabling specific depletion of endogenous SPD-5 using the dsRNA listed below. Endogenously tagged GFP::SPD-2 was generated by Cas9-induced homologous recombination [S7]. The repair template introduced an *unc-119(+)* selectable marker flanked by *loxP* sites which was subsequently removed by expressing Cre recombinase [S7]. This strain was found to be fully viable (97.4%, n=1880). Dual color strains were constructed by mating. The genotypes of all strains used are listed in the table below. All strains were maintained at 23°C.

C. elegans strains used in this study

Strain #	Genotype
DAM264	*unc-119(ed3) III; ltIs69[pAA191; Ppie-1::mcherry::spd-2; unc-119 (+)] IV; ltIs78[pKO5; Ppie-1::gfp::air-1; unc-119(+)]*
DAM276	*ltSi40 [pOD1227; Psas-6::sas-6reencoded::GFP; cb unc-119(+)] II; sas-6(ok2554) IV*
DAM309	*vieSi15[pAD395; Pspd-5::gfp::spd-5reencoded; cb unc-119(+)] II; unc-119 (ed3)] III*
DAM349	*vieSi15[pAD395; Pspd-5::gfp::spd-5reencoded; cb unc-119(+)] II; ltIs37[pAA64; Ppie-1::mcherry::his-58; unc-119 (+)] IV*
DAM640	*spd-2(vie4[spd-2::gfp +loxP]) I; ltIs37 [pAA64; Ppie-1::mcherry::his-58; unc-119 (+)]IV; ltIs44[pAA173; pie-1::mcherry::ph(plc1delta1); unc-119 (+)]*
N2	*C. elegans wild-type (ancestral)*
TH32	*unc-119(ed3) III; ruIs32[pAZ132; Ppie-1::gfp::histone h2b; unc-119(+)] III; ddIs6 [Ppie-1::gfp::tbg-1; unc-119(+)] V*

RNA-mediated interference

RNAi experiments were performed by soaking [S8] using the dsRNAs listed in the table below. Standard RNAi conditions of 48h at 16°C were used for *spd-5* to ensure full depletion. For egg shell permeabilization by *ptr-2(RNAi)*, soaking time was reduced to 9h with a recovery time of 20h at 20°C to minimize phenotypes beyond embryo permeability.

dsRNAs used in this study

Gene	Name	mg/ml	Oligo #1	Oligo #2	Template
C32E8.8	*ptr-2*	1.7	AATTAACCCTCACTAAAGGGTGGCCATCCAAGAGCTGAT	TAATACGACTCACTATAGGTGTGCCCGATCATTCTGCAT	N2 genomic DNA
F56A3.4	*spd-5*	1.9	AATTAACCCTCACTAAAGGCCGAAGACAATTTTGCCAGACA	TAATACGACTCACTATAGGTCGTGCTCAAGCTTGCTACA	N2 genomic DNA

Drug treatments

Drug treatments were performed on permeabilized embryos [S9], dissected and filmed without compression [S10] in meiosis medium containing 20μM clasto-lactacystin-ß-lactone [S9]. For arrest in metaphase of the first embryonic mitosis, embryos had to have progressed beyond meiosis II prior to exposure to the drug. Embryos already in the first mitosis tended to arrest only in the subsequent mitosis.

Immunoblots

Immunoblots were performed on whole-worm extracts with affinity-purified antibodies to SPD-5 [S11], using anti-α-tubulin (DM1α, Sigma) as a loading control.

Immunofluorescence and fixed imaging

Immunofluorescence experiments were performed as previously described [S12] using directly-labelled affinity-purified antibodies to GIP-1 [S13] and SPD-5 [S11], as well as unlabeled antibodies to α-tubulin (DM1α, Sigma). 3D widefield datasets were acquired using a 100x 1.4NA Super Plan Apochromat lens on a DeltaVision microscope equipped with a 7-Color SSI module and CoolSNAP-HQ2 cooled CCD camera, computationally deconvolved and projected using SoftWorx (Applied Precision), before being imported into Adobe Photoshop for final processing.

Live imaging

Embryos were filmed without compression [S10] under attenuated fluorescence illumination conditions known not to perturb cell cycle progression. Spinning disk confocal microscopy was performed on a Yokogawa CSU X1-A1 spinning disk confocal mounted on a Zeiss Axio Observer Z1 inverted microscope equipped with a 63x 1.4NA Plan Apochromat lens, 120mW 405nm and 100mW 488nm and 561nm

solid-state lasers, 2D-VisiFRAP Galvo FRAP module and CoolSNAP-HQ2 cooled CCD camera and controlled by VisiView software (Visitron Systems). For quantitative analysis of PCM recruitment during the normal embryonic cell cycle and during conditions of metaphase arrest, 11x1μm GFP/mCherry z-series as well as single plane DIC images were acquired every 30s from meiosis II until the end of the first mitosis or 10 minutes into mitotic arrest, using low laser illumination to minimize photobleaching. To examine protein dynamics at centrosomes during mitotic arrest, the same imaging conditions were applied. Embryos were followed from early prophase, with z-series acquired at irregular intervals. Photobleaching was performed at ~3min 30s after metaphase onset, using the galvanometer point scanner to target a region encompassing the centrosome with the 405nm laser at 10mW power. Embryos were only analysed if centrosome signal was completely eliminated throughout the entire z-volume. For spatial analysis of PCM recruitment, embryos were imaged with the additional use of a 1.6 optovar at higher laser power. Photobleaching was performed during mitotic prophase, 370-240s prior to cytokinesis onset. Embryos were then imaged until the end of the first mitosis with 12x0.3μm GFP/mCherry z-series as well as single plane DIC images acquired at irregular intervals. Partial photobleaching in metaphase-arrested embryos was performed in the same manner, except targeting the point scanner to a smaller rectangular region placed off-center to bleach only ~1/4-1/2 of the centrosome volume. Image stacks were imported into MetaMorph (analysis of PCM recruitment and protein turnover in metaphase-arrested embryos) or Fiji (spatial analysis of PCM recruitment) for post-acquisition processing.

Image analysis

PCM recruitment analysis was performed in Metamorph essentially as previously described [S6]. GFP signal was measured on single planes for both centrosomes at each time point. Images were only quantified when both centrosomes in the embryo were captured in the z-series. Two variable-size concentric boxes were drawn around each centrosome, a smaller one encompassing the centrosome, and a larger one including the surrounding cytoplasm. The integrated GFP intensity was then calculated by subtracting the mean fluorescence intensity in the area between the two boxes (mean background) from the mean intensity in the smaller box and multiplying

by the area of the smaller box. Measurements for both centrosomes at each time point were summed, compensating for centrosome movement in z within the embryo. Individual measurements from control and metaphase-arrested embryos were normalized by dividing by the mean intensity of measurements made over the interval of 30 to 90s after metaphase onset in control embryos. Recruitment curves were generated by pooling the normalized data from multiple embryos. Data points in the graphs are the mean of the normalized GFP intensity measurements collected during the 200s interval centered on that point. Error bars indicate the 90% confidence interval for the mean.

To analyze the metaphase FRAP data, the GFP signal for the bleached centrosome was quantified on single planes and the integrated fluorescence intensity calculated as described above. Measurements done after photobleaching were normalized to the mean intensity of measurements made in the 100s preceding photobleaching. The values for mobile fraction and half-life were obtained by fitting the data to the exponential equation Y=A*(1-exp(-B*X)) in GraphPad Prism, where A is the mobile fraction, and the half life $t_{1/2}$=ln(2)/B. R^2 values are the correlation coefficient obtained by fitting experimental data to the model. In cases where only a small fraction of the GFP signal recovered, it was not possible to fit the data to the model and therefore no values for mobile fraction and half-life are given. Data points on the graphs are the mean of the normalized GFP intensity measurements collected during the 10s interval centered on that point. Error bars indicate the 90% confidence interval for the mean.

To analyze the recruitment data from prophase FRAP, the fluorescence intensity distribution at each time point was quantified on single planes in Fiji. First, the center of the centrosome was identified by thresholding the image and using the “analyze particles” command to locate the center of mass. These coordinates were then used by the “radial profile 2.0” plugin to position concentric rings at 0.064μm intervals over the centrosome, spanning a total diameter of 3.7μm. The mean fluorescence intensity was then measured for each ring, as well as for the cytoplasmic background near the centrosome. Following background subtraction, measurements were then normalized to the peak intensity of the pre-bleach signal, or the peak signal at each time-point. Finally, the data was mirrored to generate a symmetrical centrosomal profile and fit to a Gaussian distribution Y=A*exp(-0.5*((X/B)^2) using GraphPad Prism, where A is

the peak intensity and B is the standard deviation. Data points on the graphs are the mean of the normalized GFP intensity measurements for each concentric region. Error bars are the 90% confidence interval for the mean. R^2 values for fitted curves were >0.95.

Predictive models for each pattern of PCM incorporation were generated by fitting the amount of protein recruited during the 65s following photobleaching (estimated based on the volume of a 2D Gaussian distribution V=2*π*A*B^2, where A is the peak intensity and B is the standard deviation) to the following curves:

- Recruitment to periphery

Y=A(t=65s)*exp(-0.5*((X/B(t=65s))^2)–A(t=0s)*exp(-0.5*((X/B(t=0s))^2)

Parameters for A(t=0s), B(t=0s, 65s) based on measurements of centrosomal signal at t=0s, t=65s (unbleached centrosome), A(t=65s) estimated from 2D volume of newly recruited protein (V) and standard deviation at t=65s (B), A=V/2*π*B^2.

- Recruitment to center:

Y=A*exp(-0.5*((X/B)^2)

Parameter for B based on measurement of metaphase centriolar SAS-6 signal, A estimated from 2D volume of newly recruited SPD-5 protein (V) and standard deviation of SAS-6 signal (B), A=V/2*π*B^2.

- Recruitment throughout pericentriolar material:

Y=A*exp(-0.5*((X/B)^2)

Parameter for B based on measurements of centrosomal signal at t=65s (unbleached centrosome), A estimated from 2D volume of newly recruited protein (V) and standard deviation at time t=65s (B), A=V/2*π*B^2.

Values used in calculating predicted profiles:

	**Measured parameters**				**Fitted parameters**		
	**SPD-5 t=0s**	**SPD-5 t=65s unbleached centrosome**	**SPD-5 t=65s bleached centrosome**	**SAS-6 metaphase**	**Periphery t=65s/0s**	**Center**	**Through-out**
Amplitude (A)	1.00	-	0.26	-	1.04/1.00	5.82	0.28
Standard deviation (B)	0.49	0.57	0.59	0.12*	0.57/0.49	0.12	0.57
Volume (V)	1.53	-	0.57	-	2.10/1.53	0.57	0.57

* SAS-6 signal is diffraction-limited, full width at half maximum based on SAS-6 distribution = 283nm.

R^2 values are the correlation coefficient obtained by fitting the experimental data obtained for bleached centrosomes at 65s to each model.

SUPPLEMENTAL REFERENCES

S1. Cabral, G., Sanegre Sans, S., Cowan, C.R., and Dammermann, A. (2013). Multiple Mechanisms Contribute to Centriole Separation in C. elegans. Curr Biol *23*, 1380-1387.

S2. Desai, A., Rybina, S., Muller-Reichert, T., Shevchenko, A., Hyman, A., and Oegema, K. (2003). KNL-1 directs assembly of the microtubule-binding interface of the kinetochore in C. elegans. Genes Dev *17*, 2421-2435.

S3. Kachur, T.M., Audhya, A., and Pilgrim, D.B. (2008). UNC-45 is required for NMY-2 contractile function in early embryonic polarity establishment and germline cellularization in C. elegans. Developmental Biology *314*, 287-299.

S4. Portier, N., Audhya, A., Maddox, P.S., Green, R.A., Dammermann, A., Desai, A., and Oegema, K. (2007). A microtubule-independent role for centrosomes and aurora a in nuclear envelope breakdown. Dev Cell *12*, 515-529.

S5. Qiao, R., Cabral, G., Lettman, M.M., Dammermann, A., and Dong, G. (2012). SAS-6 coiled-coil structure and interaction with SAS-5 suggest a regulatory mechanism in C. elegans centriole assembly. EMBO J *31*, 4334-4347.

S6. Dammermann, A., Maddox, P.S., Desai, A., and Oegema, K. (2008). SAS-4 is recruited to a dynamic structure in newly forming centrioles that is stabilized by the gamma-tubulin-mediated addition of centriolar microtubules. J Cell Biol *180*, 771-785.

S7. Dickinson, D.J., Ward, J.D., Reiner, D.J., and Goldstein, B. (2013). Engineering the Caenorhabditis elegans genome using Cas9-triggered homologous recombination. Nat Methods *10*, 1028-1034.

S8. Green, R.A., Kao, H.L., Audhya, A., Arur, S., Mayers, J.R., Fridolfsson, H.N., Schulman, M., Schloissnig, S., Niessen, S., Laband, K., et al. (2011). A high-resolution C. elegans essential gene network based on phenotypic profiling of a complex tissue. Cell *145*, 470-482.

S9. Carvalho, A., Olson, S.K., Gutierrez, E., Zhang, K., Noble, L.B., Zanin, E., Desai, A., Groisman, A., and Oegema, K. (2011). Acute drug treatment in the early C. elegans embryo. PLoS One *6*, e24656.

S10. Monen, J., Maddox, P.S., Hyndman, F., Oegema, K., and Desai, A. (2005). Differential role of CENP-A in the segregation of holocentric C. elegans chromosomes during meiosis and mitosis. Nat Cell Biol *7*, 1248-1255.

S11. Dammermann, A., Muller-Reichert, T., Pelletier, L., Habermann, B., Desai, A., and Oegema, K. (2004). Centriole assembly requires both centriolar and pericentriolar material proteins. Dev Cell *7*, 815-829.

S12. Oegema, K., Desai, A., Rybina, S., Kirkham, M., and Hyman, A.A. (2001). Functional analysis of kinetochore assembly in Caenorhabditis elegans. J Cell Biol *153*, 1209-1226.

S13. Hannak, E., Oegema, K., Kirkham, M., Gonczy, P., Habermann, B., and Hyman, A.A. (2002). The kinetically dominant assembly pathway for centrosomal asters in Caenorhabditis elegans is gamma-tubulin dependent. J Cell Biol *157*, 591-602.
